# Fabrications of the Flexible Non-Enzymatic Glucose Sensors Using Au-CuO-rGO and Au-CuO-rGO-MWCNTs Nanocomposites as Carriers

**DOI:** 10.3390/s23198029

**Published:** 2023-09-22

**Authors:** Shu-Han Liao, Kai-Yi Shiau, Fang-Hsing Wang, Cheng-Fu Yang

**Affiliations:** 1 Department of Electrical Engineering, Tamkang University, New Taipei City 251, Taiwan; shliao@gms.tku.edu.tw; 2 Graduate Institute of Optoelectronic Engineering, National Chung Hsing University, Taichung 402, Taiwan; pgeniusgod@gmail.com; 3 Department of Electrical Engineering, National Chung Hsing University, Taichung 402, Taiwan; 4 Department of Chemical and Materials Engineering, National University of Kaohsiung, Kaohsiung 811, Taiwan; 5 Department of Aeronautical Engineering, Chaoyang University of Technology, Taichung 413, Taiwan

**Keywords:** diabetes patient, flexible, non-enzymatic glucose sensor, graphene

## Abstract

A flexible, non-enzymatic glucose sensor was developed and tested on a polyethylene terephthalate (PET) substrate. The sensor’s design involved printing Ag (silver) as the electrode and utilizing mixtures of either gold–copper oxide-modified reduced graphene oxide (Au-CuO-rGO) or gold–copper oxide-modified reduced graphene oxide-multi-walled carbon nanotubes (Au-CuO-rGO-MWCNTs) as the carrier materials. A one-pot synthesis method was employed to create a nanocomposite material, consisting of Au-CuO-rGO mixtures, which was then printed onto pre-prepared flexible electrodes. The impact of different weight ratios of MWCNTs (0~75 wt%) as a substitute for rGO was also investigated on the sensing characteristics of Au-CuO-rGO-MWCNTs glucose sensors. The fabricated electrodes underwent various material analyses, and their sensing properties for glucose in a glucose solution were measured using linear sweep voltammetry (LSV). The LSV measurement results showed that increasing the proportion of MWCNTs improved the sensor’s sensitivity for detecting low concentrations of glucose. However, it also led to a significant decrease in the upper detection limit for high-glucose concentrations. Remarkably, the research findings revealed that the electrode containing 60 wt% MWCNTs demonstrated excellent sensitivity and stability in detecting low concentrations of glucose. At the lowest concentration of 0.1 μM glucose, the nanocomposites with 75 wt% MWCNTs showed the highest oxidation peak current, approximately 5.9 μA. On the other hand, the electrode without addition of MWCNTs displayed the highest detection limit (approximately 1 mM) and an oxidation peak current of about 8.1 μA at 1 mM of glucose concentration.

## 1. Introduction

Research has demonstrated that individuals with diabetes exhibit elevated glucose levels in their bodily fluids such as urine, saliva, and sweat, offering the possibility of inferring their blood glucose concentration [1]. As a result, there is an increasing need for glucose sensors that are rapid, accurate, easy to use, and cost-effective, to meet the rising demands of the growing diabetic population [2,3]. Currently, two main types of glucose sensors are employed: enzyme-based glucose sensors [4] and non-enzyme-based glucose sensors [5]. Enzymatic glucose sensors are more sensitive to specific environmental conditions, such as temperature and pH, and are prone to external influences, necessitating stricter storage and operational conditions [4]. This type of sensor is commonly used in medical devices, such as blood glucose meters, for measuring blood glucose concentrations. In contrast, non-enzymatic glucose sensors are typically more stable than enzymatic sensors and are less susceptible to environmental interference, making them potentially more reliable in practical applications [5]. This type of sensor can be employed in various fields, including the food and beverage industry and environmental monitoring, for measuring glucose concentrations. Therefore, this research primarily focuses on the development of a flexible non-enzymatic glucose sensor with a wide detection range.

Remarkable advancements have been achieved in the optimization of bio-sensors that utilize glucose oxidase. These advancements are primarily attributed to their specificity, sensitivity, and improved manufacturing techniques [6]. Researchers have extensively studied the electro-oxidation of glucose on a wide range of electrode materials, such as platinum, gold, and palladium [7,8], as well as various alloys like platinum, lead, palladium, bismuth, and rhodium [9,10]. However, despite these efforts, the current sensor electrodes still face challenges, particularly in terms of limited sensitivity and selectivity. To overcome these challenges, there is growing interest in investigating alternative electrode materials, such as manganese, cobalt, copper, zinc, and, nickel. These metals have gained attention due to their cost-effectiveness, excellent electrocatalytic activity, and capacity to facilitate electron transfer reactions at lower voltages. Research in this direction shows promising potential to improve the performance of glucose bio-sensors significantly. This could result in more accurate and reliable glucose monitoring, benefiting diabetes management and other critical biomedical applications.

Copper oxide-based glucose sensors have attracted considerable attention because of their exceptional catalytic activity and the ease of adjusting their physical and chemical properties. In the realm of electrochemical biosensors, a vital approach to enhancing charge transfer involves the development of composite materials that combine highly electrocatalytic substances with materials possessing high conductivity [11,12]. Notably, nanostructured composite materials containing Au metal oxides have shown significant improvements in electrocatalytic activity, particularly in the context of glucose detection. In this research, our main focus has been on enhancing the glucose detection sensitivity of copper oxide (CuO). To achieve this, we investigated the incorporation of gold (Au) into the CuO structure, leveraging the synergistic effects that arise from their combination. The resulting composite material demonstrated outstanding performance in accurately determining glucose levels [13]. The development of such advanced composite materials holds tremendous promise for advancing glucose sensing technology. It has the potential to create more efficient and reliable glucose biosensors, which could significantly impact the management of diabetes and other medical applications where precise glucose monitoring is essential.

These modifications have shown promising results in enhancing the overall performance of glucose sensors. Reduced graphene oxide (rGO) is a specific form of graphene obtained through the reduction of graphene oxide, a process that eliminates oxygen functional groups from the material [14]. Using rGO for glucose detection is an advanced method that can provide high sensitivity and stability. To achieve higher sensor sensitivity, researchers have explored the incorporation of graphene in the form of metal–metal oxide modifications, such as platinum–copper oxide [15], platinum–nickel oxide [16], palladium–copper [17], and zinc oxide–cobalt oxide [18]. For example, Phetsang et al. used copper-reduced graphene oxide film modified electrode for non-enzymatic glucose sensing application [19]. There are several methods for reducing graphene oxide, including electrochemical reduction, chemical reduction, and thermal reduction, each having its own advantages and disadvantages. The choice of the appropriate reduction method is critical as it not only removes oxygen functional groups but also helps in restoring the crystal structure, which may have been damaged during the synthesis of graphene oxide due to strong oxidation reactions. As a result of the proper treatment, rGO demonstrates a lower oxygen content and an improved crystal structure compared to the original graphene oxide [20]. The controlled reduction of graphene oxide to produce rGO holds immense significance in various applications, including sensor technology, energy storage, and biomedical devices. Its enhanced properties, such as improved electrical conductivity and a higher surface area, make rGO a highly sought-after material in the field of nanotechnology. As researchers continue to refine the reduction processes, the potential of reduced graphene oxide in advancing diverse technological fields is expected to grow even further, opening up new possibilities for innovation and advancement.

Another significant advancement in sensor design involves creating composite materials consisting of redox enzymes and metal nanoparticles (or nanotubes) on the electrode surface. This approach not only addresses the fundamental issue of efficient electrical communication between redox proteins and the electrode but also opens up exciting possibilities for developing miniaturized and implantable amperometric sensors. These developments hold great potential for further improving glucose sensor technology and expanding its applications in the field of biomedical devices. These advanced sensor configurations have the potential to revolutionize glucose monitoring for individuals with diabetes, offering greater convenience and accuracy in managing their blood glucose levels. A crucial component in these composite materials is reduced graphene oxide, a form of graphene with oxygen-containing functional groups removed. It provides a larger surface area, allowing for the accommodation of more Au-CuO nanoparticles. Furthermore, the inclusion of rGO, as demonstrated in previous studies [21], or the combination of rGO and multi-walled carbon nanotubes (MWCNTs) [22] has been found to significantly optimize sensor performance. This optimization occurs through the effective enhancement of electric current, resulting in a substantial boost in sensitivity. These enhancements are attributed to well-understood mechanisms that facilitate the catalysis of glucose oxidation, ultimately leading to improved sensor capabilities. The combination of these innovative materials and design strategies holds great promise in advancing the field of glucose sensing, enabling more precise and reliable glucose measurements for medical applications, research, and diagnostics. These advancements could significantly improve the quality of life for individuals with diabetes and have a profound impact on healthcare by providing better tools for glucose monitoring and management.

The one-pot synthesis method is a versatile technique in which multiple reactions are carried out sequentially within a single reaction environment, resulting in enhanced reaction efficiency. This approach has gained widespread popularity due to its ability to streamline complex separation and purification processes during post-treatment, ultimately saving time and resources [23]. The successful implementation of the one-pot synthesis method in this example showcases its efficiency in streamlining complex multi-step reactions and holds promise for the advancement of various chemical synthesis processes. This technique plays a pivotal role in the design and development of novel compounds and materials across diverse scientific disciplines. Nanotubes, owing to their unique mechanical, chemical, and electronic properties, have given rise to various novel applications and materials, such as composite materials, electronic and optoelectronic devices, nanotools, energy storage components, and sensing elements. This research also explores the impact of nanotubes on non-enzyme glucose sensing results. To achieve this, we employ the highly efficient one-pot synthesis method to fabricate the necessary nanocomposite materials in a streamlined manner. Subsequently, the obtained nanocomposite undergoes annealing at 300 °C under atmospheric conditions for a continuous 4 h duration. This annealing step is crucial for enhancing the material’s structural integrity and optimizing its catalytic properties. Following annealing, the nanocomposite is processed into a well-dispersed form, adhering to a fixed proportion, to serve as the working electrode via the printing method for non-enzymatic electrochemical glucose detection [24]. The utilization of nanotubes in this context shows great potential for advancing glucose sensing technology, leading to more efficient and reliable glucose detection methods with various applications in medical research, diagnostics, and other fields.

To ensure robustness and stability, we have selected PET (Polyethylene terephthalate) as the substrate material for the non-enzymatic glucose sensor. PET’s excellent mechanical properties make it an ideal choice for providing a durable and reliable sensor platform. Recently, Dou and Qu efficiently synthesized rGO-AuNPs-MWCNTs through a straightforward process [25]. They then incorporated this composite material with chitosan and affixed the resulting mixture onto a glassy carbon electrode. This innovative approach led to the development of a novel electrochemical sensor capable of simultaneous detection of 4-aminophenol and acetaminophen. Ahmed et al. employed a Yb_2_O_3_.CuO@rGO nanocomposite as the active substance to enhance the glassy carbon working electrode. This approach was employed to create a highly sensitive and selective electrochemical sensor for the efficient detection of ascorbic acid [26]. So far, there are few studies using Au-CuO-rGO and Au-CuO-rGO-MWCNTs nanocomposites and changing the content of MWCNTs as sensing devices. Therefore, we will demonstrate the remarkable catalytic activity of the prepared Au-CuO-rGO nanocomposites, enabling the sensor to achieve highly sensitive quantification of glucose during the electrochemical detection process. This advanced nanocomposite-based sensor holds great promise for precise and trustworthy glucose monitoring, with potential applications in diverse biomedical and healthcare domains. Ultimately, the integration of this cutting-edge technology into clinical settings could lead to better patient outcomes and enhanced quality of life for individuals with diabetes and other health conditions requiring glucose monitoring. Furthermore, nanocomposites containing MWCNTs were subsequently fabricated for comparison (Au-CuO-rGO-MWCNTs). Working electrodes and reference electrodes were created within a small area, and glucose detection was conducted.

The concentration of glucose in the blood is typically quantified in millimoles per liter (mM), which is equivalent to milligrams per deciliter (mg/dL), rather than in the micromoles (μM) range. Under normal circumstances, blood glucose concentration falls within the range of 3.9 to 6.1 millimoles per liter (or 70 to 110 milligrams per deciliter). Micromoles (μM) are usually employed for measuring substances at lower concentrations, whereas blood glucose exhibits a relatively higher concentration, making millimoles the more commonly used unit of measurement. However, certain laboratory tests or specific research endeavors may necessitate more precise concentration measurements, and in such cases, the μM range may be employed. As a result, the Au-CuO-rGO-MWCNTs compositions were printed as the electrode to investigate for their glucose detection ability across a wide range of concentrations. The study also investigates the influence of incorporating nanotubes on the glucose sensing characteristics during the fabrication process. These findings suggest that the inclusion of MWCNTs at specific levels can enhance the performance of the flexible non-enzymatic glucose sensor for detecting low concentrations of glucose. The results of the study significantly contribute to the development of more efficient and versatile glucose sensing technologies, holding great potential for advancing glucose monitoring in various applications, including medical diagnostics and personalized healthcare. This study would also demonstrate that when using Au-CuO-rGO and Au-CuO-rGO-MWCNTs nanocomposites as carriers, the tested concentration range of non-enzymatic glucose would vary from 1 mM to 0.1 μM. The results of this research would also reveal our successful fabrication of flexible non-enzymatic glucose sensors on a PET substrate, thereby advancing the goal of creating wearable devices in the future.

## 2. Experimental Procedures

**Figure 1:**
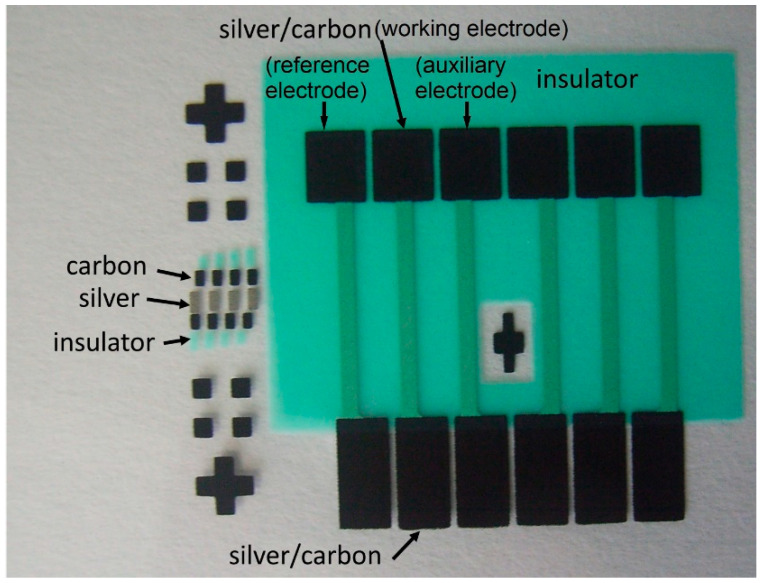
Picture of the screen-printed electrodes.

The materials used in this study were sourced from Ultimate Materials Technology Co., Ltd. (Hsinchu, Taiwan) and included high-purity substances. The key components consisted of 98% pure copper nitrate-trihydrate (Cu(NO_3_)_2_∙3H_2_O), 99.5% tetrachloroauric(III) acid trihydrate (HAuCl_4_∙3H_2_O), 99.3% sodium borohydride (NaBH_4_), and graphite oxide powder. The oxidized graphene powder had an average thickness of approximately 1 nm, with single-layer sheets ranging from 0.2 to 10 μm in diameter. Additionally, the sheet diameter varied between 500 nm and 40 μm. To facilitate the experiments, other substances were also used, including 99% glucose (glucose powder), 99% polymethyl methacrylate (PMMA), and 99% dimethylformamide (DMF). Deionized water was used consistently throughout all experimental procedures. The screen printing method was employed in this experiment to fabricate electrode and carrier materials on a flexible PET substrate, with the conductive layer comprising silver covered with carbon electrodes. Subsequently, an insulating layer was applied to cover all exposed areas, leaving only the required sensing and measurement regions exposed, as illustrated in Figure 1. The average sheet resistances of the silver electrode and the carbon electrode were measured using a four-point probe measurement technique and found to be 160 mΩ and 98 Ω, respectively. Three square electrodes were used as the test pieces, which included:

(1) Working electrode (carrier material): This electrode was printed with the nanocomposites Au-CuO-rGO or Au-CuO-rGO-MWCNTs for sensing purposes;
(2) Reference electrode: The silver/silver chloride (Ag/AgCl) glue was printed to serve as a reference electrode during sensing measurements;
(3) Auxiliary electrode: The original carbon electrode was used to assist in the electrochemical process.

To synthesize the target nanocomposite Au-CuO-rGO, we utilized a one-pot synthesis method. Initially, we subjected 200 mL of GO dispersion with a concentration of 0.5 mg mL^−1^ to ultrasonic treatment for 2 h to ensure proper dispersion in deionized water. Subsequently, under vigorous stirring at 700 rpm, we added 5.81 mL of 50 mM copper nitrate trihydrate and 0.55 mL of 50 mM tetrachloroauric(III) acid trihydrate to the GO dispersion and continued stirring for at least 30 min. For the incorporation of nanotubes, the weight ratio between GO and MWCNTs was adjusted while maintaining a concentration of 0.5 g mL^−1^, using the same experimental procedures employed for producing the GO dispersion. The ratios used for the nanocomposite synthesis were as follows: 0 wt% corresponded to 100 wt% of GO, while the 60 wt% component consisted of 40 wt% GO and 60 wt% MWCNTs. After this, 50 mL of sodium borohydride with a concentration of 0.79 M was slowly introduced into the reaction mixture and stirring at room temperature was maintained for several hours (at least 2 h). It is important to note that during this step, the solution exhibited a vigorous reaction, resulting in the production of a significant amount of gas. This process facilitated the reduction of GO to rGO while simultaneously synthesizing the desired Au-CuO-rGO nanocomposites. If MWCNTs were added, it was essential to maintain vigorous stirring throughout the entire experimental process. This is because the MWCNTs do not participate in the reaction and have a tendency to agglomerate, making it challenging to disperse them evenly throughout the reaction mixture.

After the reaction, the resulting precipitate was filtered using filter paper and washed two to three times with deionized water to remove any unreacted reactants. The obtained product was then dried in an oven at 90 °C overnight (12 h). Subsequently, the dried product was annealed in the atmosphere at 300 °C for 4 h in a furnace tube. To disperse the product obtained after annealing, it was stirred for over 3 h (300 rpm) using a magnetic stirrer to break up any agglomeration. Once it was confirmed that there were no noticeable large lumpy particles, the nanocomposite was dispersed in DMF (5 mg mL^−1^) using an ultrasonic oscillator. Before each use, the nanocomposite needed to be treated with an ultrasonic oscillator for more than 10 min to address the issue of precipitation. In this experiment, we utilized a D8 SSS X-ray diffractometer (XRD, BRUKER, Billerica, MA, USA) to analyze the structure of the nanocomposites. The surface morphology of the working electrode was examined using a S-4800 field emission scanning electron microscope (FE-SEM, HITACHI, Tokyo, Japan). This microscope confirmed the surface morphology of the nanocomposite materials, namely Au-CuO, rGO, and MWCNTs, produced during this study.

The Andor BWII RAMaker SR-750 model, a vibrational spectroscopic imaging system, was employed for Raman spectral analysis to verify the quality of the synthesized rGO and the incorporated MWCNTs. For a comprehensive elemental analysis of the samples, electron spectroscopy for chemical analysis (ESCA) was conducted using the ULVAC-PHI, PHI 5000 VersaProbe model. The experiment focused on scanning four specific elements—C, O, Au, and Cu—on each test piece. The aim was to analyze the chemical bonding composition of these elements, providing valuable insights into the characteristics of the synthesized rGO. Linear sweep voltammetry (LSV) was used as a measurement technique in which the potential varies linearly with time, enabling the quantification of the current variation with voltage [27]. During the LSV measurement, the potential between the working electrode and the reference electrode was linearly scanned while the current on the working electrode was measured simultaneously. The three-electrode system, which consisted of a working electrode, an auxiliary electrode, and a reference electrode, utilized for the measurements was the Agilent 4156C workstation. The experimental setup for linear sweep voltammetry employed a potentiostat and a three-electrode arrangement to apply a controlled potential to the solution and monitor its current response. The potentiostat, through the three-electrode configuration, provided the potential, denoted as E, which was delivered to the working electrode [28].

LSV was utilized to evaluate changes in the glucose oxidation peak currents of two types of test specimens: Au-CuO-rGO and Au-CuO-rGO-MWCNTs, with varying proportions of MWCNTs ranging from 15 wt% to 75 wt%. The glucose concentration range covered values from 0.1 μM to 10 mM, with a concentration step of 10 times for the measurements. The primary objective was to identify the concentration range in which the oxidation peak current (Ip) exhibits significant variations for different proportions of MWCNTs across the broad spectrum of glucose concentrations. Subsequently, small-scale measurements were conducted in regions with higher sensitivity, focusing on concentration ranges of 0.1 μM to 1 μM and 1 μM to 10 μM. For selective measurements, LSV was utilized to evaluate and compare the oxidation peak current for various substances, including sodium hydroxide, sodium chloride, potassium ion, urea, and glucose. The experiments were conducted in a 0.1 M sodium hydroxide solution with a glucose concentration of 1 μM. For the ampere response measurements, the CHI627D instrument was used, and a Au-CuO-40 wt% rGO-60 wt% MWCNTs test piece was employed in a 0.1 M NaOH environment. The choice of 0.1 M NaOH as the supporting electrolyte was based on its capacity to establish a stable and precisely defined electrochemical environment. The alkaline properties of NaOH guarantee a consistent pH level that is well-suited for the electrochemical reactions essential for our sensor’s operation. Two sets of measurements were performed at (a) a wide-range measurement with a 10-fold concentration increase, spanning from 0.1 μM to 1 mM and (b) amperometric response measurements within the concentration range of 0.2 mM to 1 mM.

## 3. Results and Discussion

**Figure 2:**
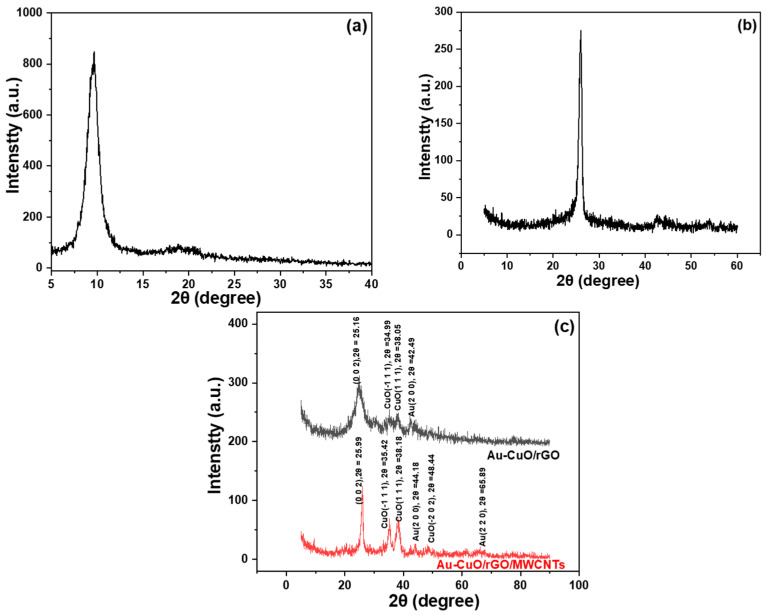
XRD diffraction patterns of (**a**) GO, (**b**) MWCNTs, and (**c**) Au-CuO-rGO and Au-CuO-40 wt% rGO-60 wt% MWCNTs.

XRD is an effective technique for material analyses and, therefore, XRD analyses were first performed on the processed materials to characterize their structures and compositions [29]. Figure 2 displays the XRD diffraction analysis results by using GO, MWCNTs, Au-CuO-rGO, and Au-CuO-40 wt% rGO-60 wt% MWCNTs as the samples for analyses. From Figure 2a, it can be observed that there is a distinct characteristic peak of GO at 2θ = 9.57°, corresponding to the preferred growth direction of (002). On the other hand, from Figure 2b, it is evident that MWCNTs exhibits a prominent peak at 2θ = 26.02° (002). However, after the synthesis of the nanocomposites, the characteristic peaks belonging to GO are significantly reduced, almost becoming invisible. This reduction in characteristic peaks of the GO can be attributed to the successful synthesis of reduced graphene oxide. In contrast, Figure 2c exhibits distinct peaks corresponding to copper oxide and gold, providing further confirmation of the successful synthesis of metal particles within the nanocomposite. The XRD analysis results are consistent with the effective reduction of GO to rGO and the presence of the desired metal particles (copper oxide and gold) in the Au-CuO-rGO nanocomposites. This discussion underscores the importance of XRD analysis in investigating structural changes and confirming the successful synthesis of the produced nanocomposites. It is worth mentioning that in the samples with the addition of MWCNTs, as shown in Figure 2c, a noticeable increase in the overall peak intensity can be observed. This enhancement could be attributed to the incorporation of MWCNTs, which reduces material agglomeration and further increases the probability of successful synthesis of copper oxide and gold. As a result, the samples with 60 wt% MWCNTs, especially in the case of copper oxide, show a significant peak enhancement.

**Figure 3:**
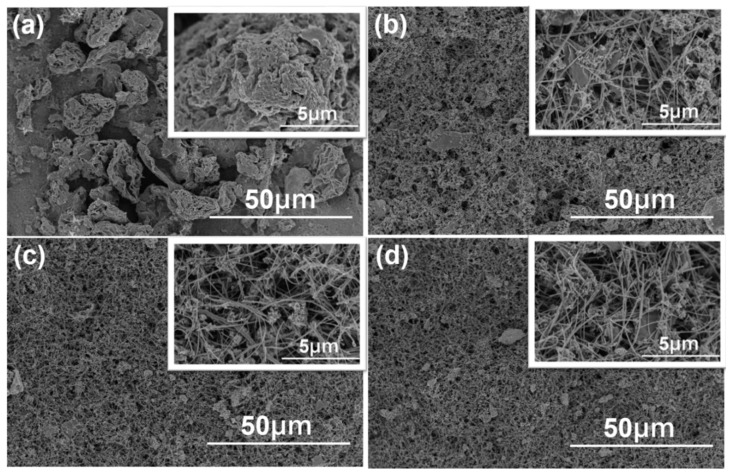
Surface SEM images of Au-CuO-rGO-MWCNTs with lower magnification for different wt% of MWCNTs (**a**) 0 wt%, (**b**) 15 wt%, (**c**) 30 wt%, and (**d**) 60 wt%. Surface SEM images of Au-CuO-rGO-MWCNTs with higher magnification are added in the inset of each image.

Figure 3 utilizes FESEM (field emission scanning electron microscopy) to examine the surface morphology of test specimens with varying weight percentages of MWCNTs, ranging from 0 wt% to 60 wt%. In Figure 3, a low-magnification FESEM image is presented, while a high-magnification view is provided in the inset. Clearly, the incorporation of MWCNTs has a significant impact on the overall surface morphologies. In Figure 3a, the surface morphology of the 0 wt% sample reveals the presence of only agglomerated rGO. However, with the addition of 15 wt% MWCNTs, as Figure 3b shows, the primary structure transforms from a granular sheet form into a linear arrangement of carbon nanotubes. Additionally, as depicted in the inset of Figure 3b, modified metal nanoparticles are observed on the surfaces of the linear MWCNTs. It is evident that in the sample without MWCNTs (0 wt%), the nanocomposite materials do not entirely cover the surface of the test piece, likely due to the relatively low concentration of the dispersed liquid used during the synthesis process. As the proportion of MWCNTs increases, as depicted in Figure 3c for samples with 30 wt% MWCNTs addition, and Figure 3d for sample with 60 wt% MWCNTs addition, it becomes evident that the volume occupied by MWCNTs is significantly greater than that of rGO, even when the mass is the same. As a result, even with the same dispersion liquid ratio, the entire test piece surface can be completely covered. Moreover, the incorporation of MWCNTs effectively reduces the agglomeration of rGO. These experimental findings provide initial confirmation that this reduction in agglomeration contributes to an increased success rate in synthesizing copper oxide and gold.

**Figure 4:**
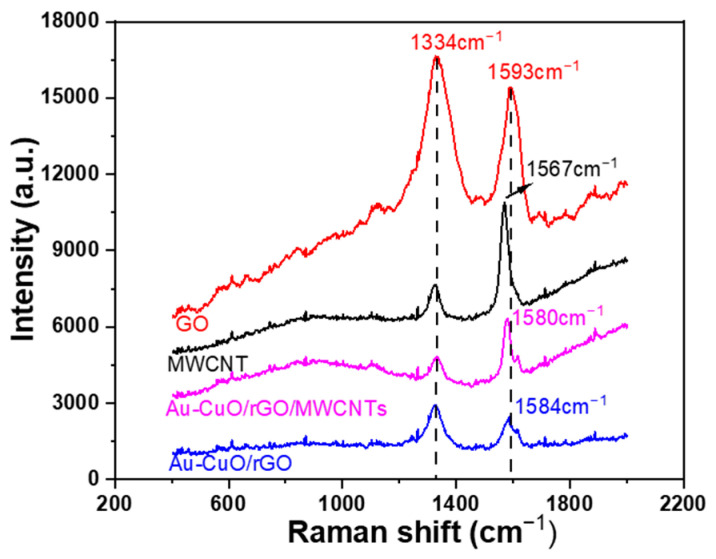
Raman full spectrum for GO, MWCNTs, Au-CuO-rGO, and Au-CuO-40 wt% rGO-60 wt% MWCNTs.

Raman spectroscopy is a powerful technique widely used to investigate the vibrational properties of graphene [16]. It often exhibits two prominent vibrational modes known as the G and D bands. The G band originates from the first-order scattering of the E2g phonon associated with the hexagonal arrangement of sp2 carbon atoms. On the other hand, the D band is attributed to the breathing mode of the A1g symmetry phonon at the K point [28]. In this study, the Raman spectra of different graphene-based materials were examined, and the results are shown in Figure 4. GO displayed a D band at 1334 cm^−1^ and a G band at 1593 cm^−1^, while for MWCNTs, the bands were located at 1328 cm^−1^ and 1567 cm^−1^. For the Au-CuO-rGO nanocomposite, the spectral peaks were observed at 1328 cm^−1^ and 1584 cm^−1^, and for the Au-CuO-rGO-MWCNTs composite showed Raman bands at 1333 cm^−1^ and 1580 cm^−1^. The blue shift of the G band observed in these materials can be attributed to the reduction in oxygen functionalities during the reduction process. Notably, the Raman spectrum of Au-CuO-rGO exhibited an increased I_D_/I_G_ ratio (I_D_ and I_G_ are the intensities at D band and G band), which suggests that the formation of rGO led to a further increase in defects. Furthermore, the incorporation of MWCNTs in the Au-CuO-rGO-MWCNTs composite contributed to an additional blue shift of the G band. The specific I_D_/I_G_ ratios for GO, MWCNTs, Au-CuO-rGO, and Au-CuO-40 wt% rGO-60 wt% MWCNTs were 1.09, 0.594, 1.25, and 0.760, respectively. The observed variations in the Raman spectra and the calculated I_D_/I_G_ ratios provide valuable insights into the structural and chemical changes occurring in the graphene-based materials due to different processing methods and the incorporation of nanotubes. Such information can be crucial for tailoring the properties of graphene-based composites for various applications, such as electronics, energy storage, and catalysis. However, further comprehensive analysis and characterization are necessary to fully understand the underlying mechanisms responsible for these spectral shifts and their impact on the material’s performance.

**Figure 5:**
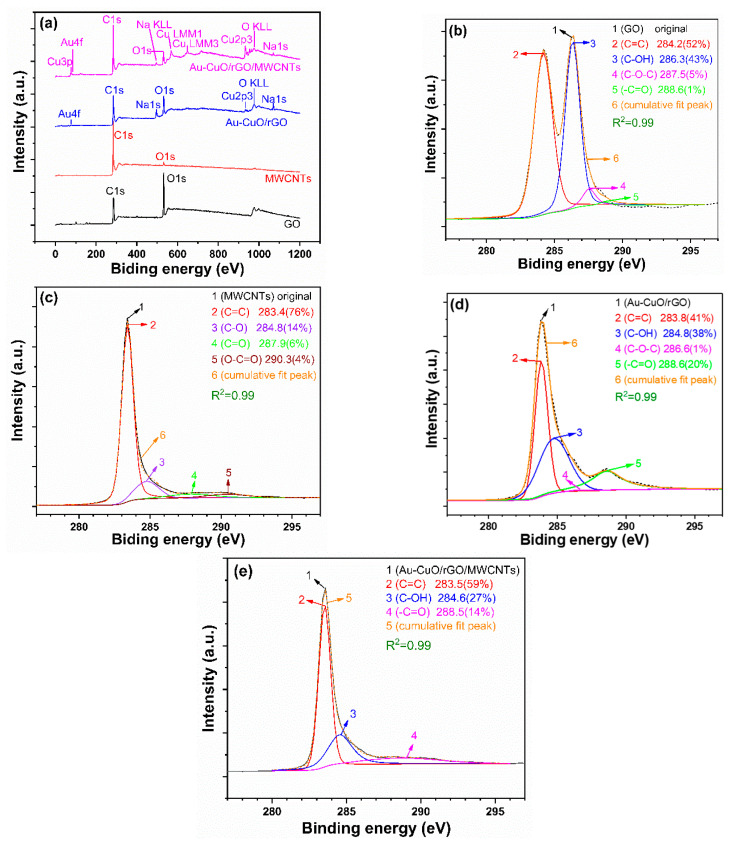
XPS analyses for (**a**) full spectrum of C1s peak spectrum, (**b**) GO, (**c**) MWCNTs, (**d**) Au-CuO-rGO, and (**e**) Au-CuO-40 wt% rGO-60 wt% MWCNTs.

XPS is a valuable analytical technique employed for studying the elemental composition and chemical states of materials [30]. Together with other characterization techniques, XPS plays a critical role in revealing essential information about nanomaterials, assisting in their design and optimization for specific applications. The mitigation of the charging effect in XPS and the subsequent correction of binding energy (BE) data related to C=O (carbon-oxygen) bonds can be accomplished through various methods. In our study, we employed the background subtraction method to rectify the BE data associated with C=O bonds. Charging phenomena in XPS manifest when the sample exhibits insulating properties or possesses low electrical conductivity. During such instances, electrons are either removed from or added to the sample surface, resulting in a deviation of the observed binding energies. To counteract the charging effect, a commonly employed strategy involves subtracting the background signal generated by the charging process. This background signal can be ascertained by measuring the emission of electrons from an inert reference material, such as a metallic foil, positioned in proximity to the sample. The disparity in binding energy between the reference material and the sample can then be employed to correct the shifts induced by charging. In this particular case, the spectra display the binding energy of carbon (C1s) in various samples, offering insights into the types of carbon bonds present. Figure 5a shows the XPS spectra of GO, MWCNTs, Au-CuO-rGO, and Au-CuO-40 wt% rGO-60 wt% MWCNTs samples.

Figure 5b–e depicts the C1s peak spectra in the range of 275 eV to 300 eV for different samples. In Figure 5b, the rGO peaks correspond to C=C (284.2 eV), C-OH (286.3 eV), C-O-C (287.5 eV), and -C=O (288.6 eV). Figure 5c shows the MWCNTs peaks corresponding to C=C (283.4 eV), C-O (284.8 eV), C=O (287.9 eV), and O-C=O (290.3 eV). In Figure 5d, the peaks for Au-CuO-rGO are C=C (283.8 eV), C-OH (284.8 eV), C-O-C (286.6 eV), and -C=O (288.6 eV). Lastly, Figure 5e presents the peaks for Au-CuO-40 wt% rGO-60 wt% MWCNTs, which are C=C (283.5 eV), C-OH (284.6 eV), and -C=O (288.5 eV). The goodness of fit parameter (R-squared value) is also shown in the figure. From the XPS spectra, the introduction of rGO and the addition of MWCNTs lead to peak trends approaching those of MWCNTs. Specifically, the intensity of the C-OH bond in GO significantly decreases, while the intensity of the C=C bond characteristic of MWCNTs noticeably increases. Comparing GO, MWCNTs, Au-CuO-rGO, and Au-CuO-40 wt% rGO-60 wt% MWCNTs samples highlights the changes in chemical bonding when different materials are combined. The incorporation of rGO and MWCNTs appears to influence the chemical environment, resulting in similarities between the peaks observed for Au-CuO-40 wt% rGO-60 wt% MWCNTs and MWCNTs. This suggests possible interactions between rGO, MWCNTs, and the Au-CuO nanoparticles, affecting the electronic structure and bond configurations of the resulting nanocomposites.

**Figure 6:**
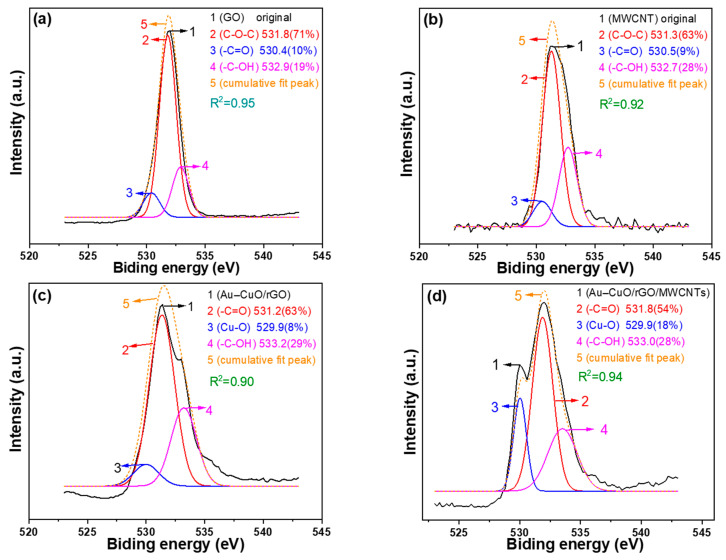

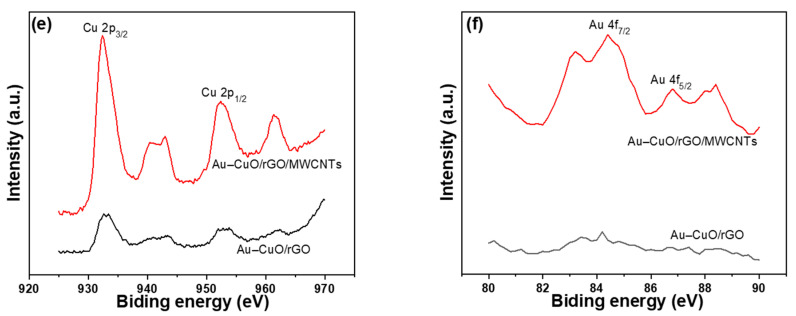
XPS analyses for O1s peak spectra of (**a**) GO, (**b**) MWCNTs, (**c**) Au-CuO-rGO, (**d**) Au-CuO-40 wt% rGO-60 wt% MWCNTs, and for (**e**) Cu 2p and (**f**) Au 4f.

To further investigate the influence of the materials used on their properties, we conducted X-ray photoelectron spectroscopy (XPS) analysis to determine the oxygen chemical bonding state, and Figure 6a–d display the deconvoluted O1 spectra in the 523–543 eV range. In Figure 6a, the peaks correspond to different oxygen species in rGO at 531.8 eV for C-O-C, 530.4 eV for -C=O, and 532.9 eV for -C-OH. In Figure 6b, the peaks correspond to oxygen species in the MWCNTs at 531.3 eV for C-O-C, 530.5 eV for -C=O, and 532.7 eV for -C-OH. Figure 6c shows the peaks of Au-CuO-rGO, with peaks at 531.2 eV for -C=O, 529.9 eV for Cu-O, and 533.2 eV for -C-OH. Finally, in Figure 6d, the peaks for Au-CuO-rGO-MWCNTs are observed at 531.8 eV for -C=O, 529.9 eV for Cu-O, and 533.0 eV for -C-OH. Figure 6e displays the Cu 2p spectra, with a distinct Cu 2p peak at 930 eV, indicating the presence of metallic copper. Another peak near 945 eV corresponds to Cu^2+^ species, confirming the successful synthesis of CuO. Figure 6f shows the Au 4f spectra, with peaks presented at 84.4 eV and 86.8 eV corresponding to Au 4f_7/2_ and Au 4f_5/2_, respectively, which indicate the presence of gold in the sample. These results prove that with the addition of MWCNTs, the emission intensity of the Cu-O bond in the O1 peak spectrum significantly increases, further validating that the addition of MWCNTs enhances the successful synthesis of CuO. This suggests that the addition of MWCNTs plays a crucial role in improving the characteristics of the CuO composite, potentially leading to enhance the performance of the Au-CuO-rGO-MWCNTs in the application of sensing the non-enzymatic glucose.

**Figure 7:**
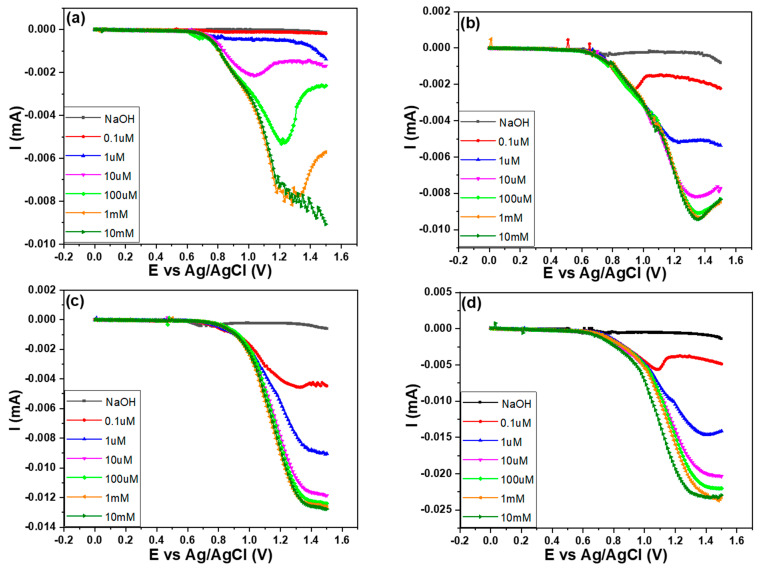
Glucose LSV measurements for samples with different proportions of MWCNTs, (**a**) 0 wt%, (**b**) 15 wt%, (**c**) 30 wt%, and (**d**) 60 wt%.

The study utilized the LSV method ranging from 0 V to 1.5 V to investigate the electrochemical response of glucose in different concentrations, without and with the different contents of MWCNTs addition. The measurements were conducted under a 0.1 M NaOH environment, and the detection limit is the concentration at which the sensor can reliably detect the presence of glucose and provide a measurable signal. From Figure 7a, it can be observed that for the Au-CuO-rGO sample, the detection limit was relatively high. When the concentration of glucose reaches 1 mM, the Au-CuO-rGO sensor responds obviously. This indicates that the sensor’s sensitivity was limited when there was no addition of MWCNTs, as it required a relatively high glucose concentration to produce a detectable signal. Even at the lowest concentration of 0.1 μM, there was a slight increase in current, but the oxidation peak was not really observed. The appearance of a clear oxidation peak at 10 μM suggests that the sensor’s performance is improved at higher concentrations. In Figure 7b–d, as the proportion of MWCNTs in the Au-CuO-rGO-MWCNTs samples increased, the oxidation peak for 0.1 μM glucose concentration became much more prominent. Furthermore, the increase in current amplitude decreased significantly after reaching approximately 10 μM concentration. These observations suggest that the inclusion of MWCNTs in the Au-CuO-rGO-MWCNTs samples enhanced the sensors’ sensitivity for detecting lower glucose concentrations while attenuating the response at higher concentrations. This heightened sensitivity at low concentrations is extremely valuable, as it allows the sensor to detect even minute amounts of glucose. The study’s findings emphasize the importance of integrating MWCNTs to enhance the sensitivity of glucose detection, especially at low concentrations. By incorporating MWCNTs, the overall performance of the sensor is improved, resulting in reduced detection limits and increased efficiency in detecting glucose at lower concentrations. However, the research also indicates a potential trade-off between sensitivity at low concentrations and the response at higher concentrations. These valuable insights can be utilized to optimize glucose biosensors for various applications, such as medical diagnostics and glucose monitoring for individuals with diabetes. By leveraging the benefits of MWCNTs, researchers and developers can design more effective and accurate glucose sensors to better cater to the diverse needs of users in the medical field.

**Figure 8:**
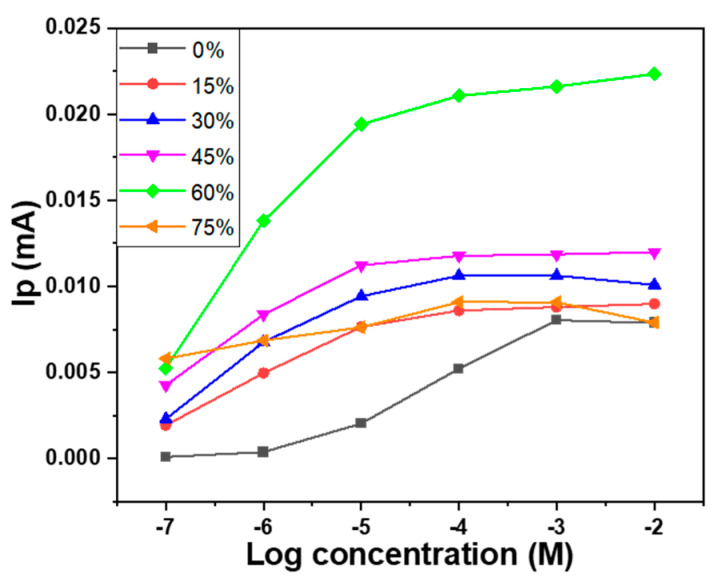
Peak currents of the fabricated sensors measured at different glucose concentrations and with different proportions of MWCNTs. Ip: peak current.

The trends of the change in peak current slope with increasing glucose concentration in a wide range of measurements are observed, and the results are shown in Figure 8. For the sample with 0 wt% MWCNTs, the current shows a more significant variation in the glucose concentration range of approximately 10 µM to 100 µM. As the percentage of the MWCNTs increases, it is evident that the range with higher slope shifts towards lower concentrations, moving from the range of 10 µM to 100 µM for the sample with 0 wt% MWCNTs to 0.1 µM to 10 µM for the sample with 60 wt% MWCNTs. This shift can be attributed to the introduction of MWCNTs, which reduces the resistance of the Au-CuO-rGO nanocomposite responsible for sensing glucose but significantly enhances the current signal for low glucose concentrations. Consequently, the detection limit for high glucose concentrations is significantly reduced to around 10 µM. Additionally, the detection of glucose at the lowest concentration of 0.1 µM becomes much more distinct compared to the Au-CuO-rGO sample. Figure 7 and Figure 8 clearly illustrate the sensitivity comparison with different proportions of MWCNTs. Among the various concentrations tested, the Au-CuO-40 wt% rGO-60 wt% MWCNTs sample displayed the optimal sensitivity within the lowest concentration range of 0.1 µM to 1 µM. On the other hand, for the Au-CuO-rGO sample, the best glucose sensitivity was observed in the range of 10 µM to 100 µM. While there were slight variations in current magnitude among the samples, the incorporation of MWCNTs significantly enhanced the overall current response. Notably, the Au-CuO-40 wt% rGO-60 wt% MWCNTs sample exhibited the highest sensitivity to low glucose concentrations, large peak currents, and overall stability.

The possible reason for the higher sensing efficiency of the Au-CuO-40 wt% rGO-60 wt% MWCNTs sample is that MWCNTs have a higher surface area. The surface area of MWCNTs plays a crucial role in their sensing performance, particularly in non-enzymatic glucose detection. MWCNTs possess a unique cylindrical nanostructure, which results in a high surface area providing numerous active sites for glucose molecules to adsorb and react. As the surface area increases, the capacity to accommodate glucose molecules also rises, leading to heightened sensitivity. Moreover, the elevated surface area allows MWCNTs to adsorb a larger quantity of glucose molecules, promoting enhanced electron transfer between the glucose molecules and the MWCNT surface. This improved electron transfer significantly boosts the sensitivity of glucose detection. Additionally, the abundance of available sites on a larger surface area facilitates glucose oxidation reactions, leading to a lower detection limit for the sensor. Consequently, the sensor becomes more sensitive, capable of detecting even lower concentrations of glucose. Although higher surface area generally improves sensitivity, it also contributes to increased stability and reusability of MWCNT-based sensors. The surplus of active sites enables repeated glucose sensing without experiencing a significant loss in performance. This potential for extended use enhances the practical applicability of MWCNT-based glucose sensors.

On the other hand, the power sensing characteristics of the Au-CuO-25 wt% rGO-75 wt% MWCNTs sample may be attributed to the excessive addition of MWCNTs. An excessive amount of MWCNTs can lead to a decrease in sensitivity in a glucose sensor made with MWCNTs due to several factors:

(a) Hindered mass transport: When the content of MWCNTs in the composition of Au-CuO-rGO-MWCNTs is too high, it can result in a densely packed and compacted structure, which hinders the diffusion of glucose molecules to the active sensing sites on the MWCNTs. As a consequence, this reduced mass transport restricts the number of glucose molecules that can interact with the sensor’s surface, thereby decreasing its sensitivity to glucose detection.
(b) Limited surface area: An elevated MWCNTs content in the composition of Au-CuO-rGO-MWCNTs can result in a reduced effective surface area available for glucose molecules to interact with. Consequently, fewer glucose molecules can be captured and detected by the sensor, leading to a decrease in sensitivity.
(c) Electron transfer inefficiency: In glucose sensors, the efficient interaction between glucose and MWCNTs depends on smooth electron transfer between them. An excessive amount of MWCNTs in the composition of Au-CuO-rGO-MWCNTs can disrupt this electron transfer process, resulting in a diminished response to changes in glucose concentration.
(d) Non-specific binding: A high content in the composition of Au-CuO-rGO-MWCNTs may increase the likelihood of non-specific binding with other interfering substances present in the sample. This non-specific binding can lead to inaccurate readings and diminish the sensor’s sensitivity and selectivity to glucose.

To maintain optimal sensitivity in a glucose sensor made with the composition of Au-CuO-rGO-MWCNTs, it is crucial to carefully optimize the proportion of MWCNTs to strike a balance between surface area, electron transfer efficiency, and mass transport, while minimizing non-specific interactions with other substances. As a result, at a glucose concentration of 0.1 µM, the sample exhibits the maximum Ip value. However, with increasing glucose concentration, there is no significant increase in the Ip value.

**Figure 9:**
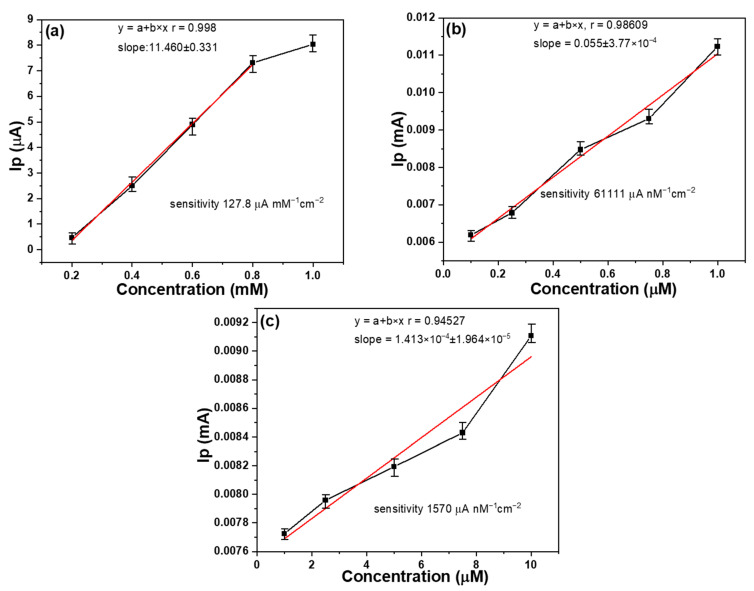
Linear characteristics of different sensors at different glucose concentrations (**a**) sample with Au-CuO-rGO as the sensor and in the range of 0.2 mM~1 mM, using Au-CuO-40 wt%rGO-60 wt% MWCNTs as the sensor and in the range of (**b**) 0.1 μM~1 μM and (**c**) 1 μM~10 μM. Ip: peak current.

Sensitivity and selectivity were measured using linear sweep voltammetry (LSV) on samples with the additions of 0 wt% and 60 wt% MWCNTs, and the ability to detect glucose was determined based on peak current. The scan range for LSV measurements ranged from 100 mV/s to 1000 mV/s, with a 0.1 M sodium hydroxide solution. For each type of sample, we prepared five replicates, and each of these samples underwent two separate tests. The average results of these tests are depicted in Figure 9, and furthermore, the overall deviation range of the testing is represented using error bars in Figure 9 as well. From the error bars in the figure, it can be observed that the samples prepared for this study exhibit acceptable and reliable stability and uniformity. This demonstrates the robustness and consistency of the samples used in our research. For the sample containing 0 wt% MWCNTs, as depicted in Figure 9a, the detection limit was achieved at approximately 1 mM concentration, and fell within the range of 0.2 mM to 0.8 mM. The sensitivity of the fabricated device was calculated by dividing the measured oxidation peak current by the glucose concentration and then further dividing it by the working electrode area (2.4 mm × 3.0 mm), and 127.8 μA nM^−1^ cm^−2^ was obtained. In the range of 0.02 mM to 0.1 mM, although there was a clear oxidation peak, the linearity was poor, preventing further analysis (not shown here). As depicted in Figure 9b, for the sample with 60 wt% MWCNTs, a significant change in peak current was observed within the glucose concentration range of 0.1 μM to 1 μM, with a high sensitivity of 61,111 μA nM^−1^ cm^−2^. In Figure 9c for the sample with 60 wt% MWCNTs, as the concentration was changed from 1 μM to 10 μM, the change in peak current was relatively smaller. However, there was still a high sensitivity of 1570 μA nM^−1^ cm^−2^.

One crucial evaluation parameter is the coefficient of determination, represented by the R-squared (R^2^) value, commonly referred to as R squared. In the context of this study, various sensors were employed to detect different glucose concentrations, and their corresponding R-squared values for the results in Figure 9a–c were calculated as 0.998 (within the range of 0.2 mM to 0.8 mM), 0.986, and 0.945, respectively. The high R-R values for the sensors with different proportions of MWCNTs and measured at different concentrations of glucose reveal their linear characteristics. These linear characteristics make these manufactured sensors highly valuable for detecting glucose. The 3σ method listed below can be used to determine the limit of detection (LOD) of glucose [31]:

LOD = 3 standard deviation/slope (1)

For the sample with 60 wt% MWCNTs, the detection limit was approximately 0.1 μM within the concentration range of 0.1 μM to 1 μM, while for the sample with 0 wt% MWCNTs, it was approximately 0.08 mM within the concentration range of 0.2 mM to 1 mM.

The study aimed to evaluate the sensitivity and selectivity of glucose detection using the LSV measurements. This research used samples with the additions of 0% and 75 wt% MWCNTs to compare their responses to the effects of different glucose concentrations on their peak current responses. From the results, it can be observed that the Au-CuO-rGO sample exhibited good sensitivity and linearity within specific concentration ranges. However, its performance was limited for lower concentrations (0.02 mM to 0.1 mM), making it less accurate in detecting glucose at such low levels. On the other hand, the Au-CuO-40 wt% rGO-60 wt% MWCNTs samples demonstrated higher sensitivity within the concentration range of 0.1 μM to 1 μM and lower sensitivity within the range of 1 μM to 10 μM, suggesting an improvement due to the addition of appropriate MWCNTs. This study shows that the sensor is most effective for glucose concentrations within the micromolar range (0.1 μM to 1 μM), with excellent linearity and sensitivity. Additionally, the sensitivity of the Au-CuO-40 wt% rGO-60 wt% MWCNTs sample is significantly higher than that of the Au-CuO-rGO sample. The variation in peak current with glucose concentration and the achieved sensitivities provide valuable information on the detection capability of the fabricated sensors. However, as the glucose concentration increases beyond this range (up to 10 μM), the change in peak current becomes less pronounced, although the sensor still maintains reasonable linearity and sensitivity. Overall, this study highlights the glucose concentration and the use of different proportions of MWCNTs in determining the performance of the sensors for glucose detection. These findings contribute to the development of glucose biosensors and other electrochemical sensing applications.

**Figure 10:**
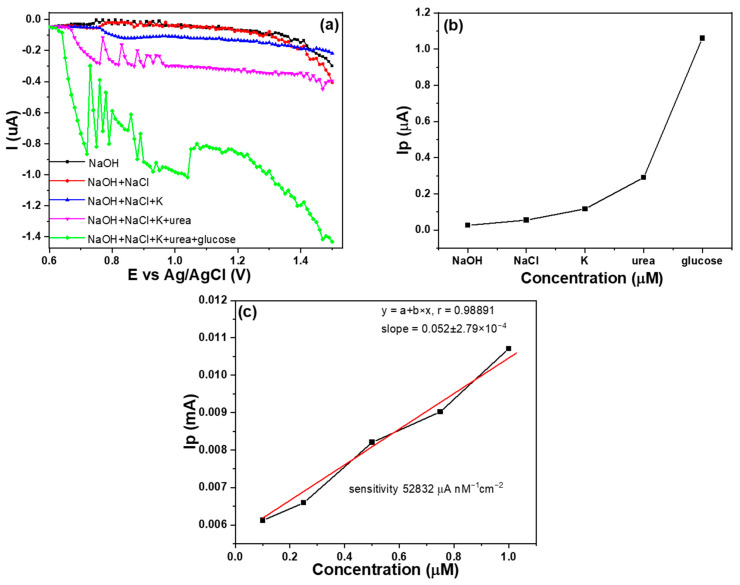
(**a**) Glucose selectivity of LSV measurements, (**b**) peak current comparison of different additives, and (**c**) Au-CuO-40 wt%rGO-60 wt% MWCNTs as the sensor for the measurements of NaOH (0.5 M) + glucose.

Due to the electrode containing 60 wt% MWCNTs exhibiting a significantly higher response compared to other electrodes, it was selected for the selective analysis. The measurements were carried out for substances like sodium hydroxide, sodium chloride, potassium ions, urea, and glucose by sequentially adding them one by one. The variation of current with voltage for different compositions on Ag/AgCl is illustrated in Figure 10a. Moreover, Figure 10b shows the comparison of peak currents resulting from the continuous addition of different substances at a concentration of 1 μM in an environment of 0.1 M sodium hydroxide. As depicted in Figure 10, it can be observed that, except for glucose, the other added substances exhibited some response, but their increase in peak current compared to glucose was not significant. These results emphasize the high selectivity of the electrode with 60 wt% MWCNTs. The experimental results show that glucose exhibited the most substantial increase in peak current among the tested substances, indicating a more pronounced electrochemical response. This behavior can be attributed to the distinct chemical characteristics and redox properties of glucose, distinguishing it from the other substances. The experimental results for the measurements of NaOH (0.5 M) + glucose (ranging from 0.1 to 1.0 μM) have been included in Figure 10c, demonstrating a high sensitivity of 52,832 μA nM^−1^ cm^−2^.

The use of MWCNTs instead of rGO in Au-CuO-rGO-MWCNTs glucose sensors can significantly enhance the sensing effect for several reasons:

(a) High surface area: MWCNTs offer a large surface area, providing numerous active sites for glucose molecules to interact with the sensor. This increased surface area enables a higher number of glucose molecules to be adsorbed, leading to improved sensitivity and detection capabilities.
(b) Excellent electrical conductivity: MWCNTs exhibit outstanding electrical conductivity, facilitating efficient electron transfer during the glucose sensing process. This enhanced electron transfer ensures the faster and more accurate response of the sensor to changes in glucose concentration. As a result, the sensor can rapidly detect variations in glucose levels with greater precision.
(c) Synergistic effects: The incorporation of Au-CuO along with both rGO and MWCNTs creates synergistic effects that enhance the overall performance of the sensor. The Au-CuO nanoparticles can enhance the catalytic activity towards glucose oxidation, while rGO and MWCNTs improve the electrical properties and stability of the sensor, resulting in more efficient and reliable glucose detection.
(d) Enhanced stability and durability: MWCNTs are known for their exceptional mechanical strength and chemical stability, which significantly improves the sensor’s overall stability and durability. As a result, the sensor can maintain its sensing capabilities over an extended period without experiencing degradation.
(e) Versatility: MWCNTs can be easily functionalized with various chemical groups, allowing for the modification of surface properties and the customization of the sensor’s selectivity towards glucose. This versatility enables the detection of glucose in complex biological samples without interference from other substances, making the sensor highly adaptable and practical.

In comparison to other studies on glucose sensors, the use of Au-CuO-rGO and Au-CuO-rGO-MWCNTs nanocomposites as carriers in the fabrication of flexible non-enzymatic glucose sensors offers several distinct advantages:

(a) Heightened Sensitivity: these nanocomposites exhibit the capacity to significantly enhance the sensitivity of non-enzymatic glucose sensors. The inclusion of Au, CuO, rGO, and MWCNTs results in an exceptionally conductive and catalytically active surface for glucose oxidation, thereby leading to improved sensor performance.
(b) Swift Response Time: the distinctive properties of these nanocomposites facilitate the rapid detection of glucose, yielding a swift response time. This characteristic is particularly vital for applications that necessitate real-time monitoring.
(c) Adaptability: the capability to fabricate these sensors on flexible substrates endows them with adaptability and versatility, rendering them suitable for various shapes and applications, such as wearable devices and biomedical sensors.
(d) Interference Mitigation: non-enzymatic sensors constructed with these nanocomposites demonstrate reduced susceptibility to interference from substances other than glucose, resulting in more precise measurements, even within complex sample matrices.
(e) Broad Detection Range: non-enzymatic glucose sensors employing Au-CuO-rGO-MWCNTs nanocomposites as carriers may boast a wide detection range for glucose concentrations when compared to enzymatic sensors.

Nevertheless, certain disadvantages should be considered:

(a) Cost Factor: the fabrication of Au-CuO-rGO and Au-CuO-rGO-MWCNTs nanocomposites can be relatively costly due to the incorporation of precious metals like gold and additional materials such as carbon nanotubes. This expense may pose a limitation on their widespread adoption.
(b) Technical Complexity: the synthesis and integration of these nanocomposites can be technically intricate and demand specialized equipment and expertise.

## 4. Conclusions

The analysis results of Raman spectroscopy showed that the incorporation of MWCNTs in the Au-CuO-rGO-MWCNT composite contributed to a blue shift of the G band, and the specific I_D_/I_G_ ratios for GO, MWCNTs, Au-CuO-rGO, and Au-CuO-40 wt% rGO-60 wt% MWCNTs were 1.09, 0.594, 1.25, and 0.760, respectively. The analysis results of XPS spectroscopy showed that the addition of MWCNTs plays a crucial role in improving the characteristics of the CuO composite and leading to enhance the sensing performance of the Au-CuO-rGO-MWCNTs in the non-enzymatic glucose. The detection limit of the Au-CuO-rGO sample was achieved at approximately 1 mM concentration, and falling, and it had a sensitivity of 127.8 μA nM^−1^ cm^−2^ within the range of 0.2 mM to 0.8 mM. When the Au-CuO-40 wt% rGO-60 wt% MWCNTs sample was used, the high sensitivity of 61,111 μA nM^−1^ cm^−2^ was observed within the glucose concentration range of 0.1 μM to 1 μM and the high sensitivity of 1570 μA nM^−1^ cm^−2^ was observed within the glucose concentration range of 1 μM to 10 μM. When the content of MWCNTs increases from 0 to 60 wt%, it is evident that the sensitivity of the fabricated Au-CuO-rGO-MWCNTs non-enzymatic glucose sensor is significantly enhanced. In other words, it allows for the detection of concentrations in the micromolar (μM) range, as opposed to the millimolar (mM) range. This improvement in sensitivity suggests that the sensor becomes more capable of detecting lower concentrations of glucose in a sample, making it more suitable for applications requiring high precision and detection of trace amounts of glucose. The sample containing Au-CuO-40 wt% rGO-60 wt% MWCNTs also exhibited strong selectivity in detecting glucose, even in the presence of interfering substances such as sodium hydroxide, sodium chloride, potassium ions, and urea. This indicates that the sensor can accurately distinguish glucose from other compounds, making it reliable for practical applications where such interferences might occur. In summary, the addition of MWCNTs to the Au-CuO-rGO-MWCNTs glucose sensors brings about significant enhancements in sensitivity, stability, and versatility. These improvements make the sensors more effective in their ability to detect glucose accurately and with high precision, even in complex sample matrices.

## Data Availability

Not applicable.
